# Effect of external airflow resistive load on postural and exercise-associated cardiovascular and pulmonary responses in pregnancy: a case control study

**DOI:** 10.1186/s12884-015-0474-7

**Published:** 2015-02-22

**Authors:** Jung-Hyun Kim, Raymond J Roberge, Jeffrey B Powell

**Affiliations:** National Personal Protective Technology Laboratory, National Institute for Occupational Safety and Health, Centers for Disease Control and Prevention, 626 Cochrans Mill Road, Pittsburgh, 15236 PA USA

**Keywords:** Pregnancy, External airflow resistive loads, Effects, Cardiovascular, Pulmonary

## Abstract

**Background:**

Facial coverings (e.g., balaclavas, niqabs, medical/surgical masks, respirators, etc.), that impose low levels of airflow resistive loads, are worn by millions of pregnant women worldwide, but little data exist addressing their impact on pregnancy-associated cardiovascular and pulmonary responses.

**Methods:**

16 pregnant and 16 non-pregnant women were monitored physiologically (heart rate, blood pressure, mean arterial pressure, total peripheral resistance, stroke volume, cardiac output, oxygen saturation, transcutaneous carbon dioxide, fetal heart rate) and subjectively (exertion) for 1 h of mixed sedentary postural activity (sitting, standing) and moderate exercise (bicycle ergometer) with and without wearing N95 filtering facepiece respirators with filter resistive loads of 94.1 Pa (9.6 mm H_2_O) – 119.6 Pa (12.2 mm H_2_O) pressure.

**Results:**

The external airflow resistive loads were associated with increases in diastolic pressure (p = 0.004), mean arterial pressure (p = 0.01), and subjective exertion score (p < 0.001) of all study subjects. No significant differences were noted with the external resistive loads between the pregnant and non-pregnant groups for any cardiovascular, pulmonary and subjective variable over 1 h.

**Conclusions:**

Low external airflow resistive loads, during combined sedentary postural activity and moderate exercise over 1 h, were associated with increases in the diastolic and mean arterial pressures of all study subjects, but pregnancy itself was not associated with any significant differences in physiologic or subjective responses to the external airway resistive loads utilized in the study.

## Background

Worldwide, many women wear various types of facial coverings that cover the nose and mouth (e.g., veils, balaclavas, bandanas, niqabs, burqas, respirators and medical/surgical masks, cloth masks, etc.) for any of a number of purposes (e.g., heat/moisture exchangers in cold environments, protection from airborne contaminants and infectious agents, religious/social norms, etc.) [1-7]. These facial coverings impose variable external resistive loads (~19.6 – 196.1 Pa [2 – 20 mm H_2_O] pressure) to the wearer, depending on fabric properties (e.g., fiber diameter, packing density, pore size, electrostatic charge, etc.) [4,5,8] and how tightly the covering is applied to the face, that have been shown to negatively impact pulmonary function and cardiovascular responses [1,6]. Given that an estimated 208 million pregnancies occur worldwide annually [9], a significant proportion of women wearing facial coverings will be pregnant, yet little scientific data is available on the effects of these low external airflow resistive loads (EARL) on pregnancy-associated cardiovascular and pulmonary responses [10,11]. The current study, part of a larger U.S. government interagency working group effort (Project BREATHE) [12] that is examining the effects of respiratory protective equipment on wearers and some of the results of which have been previously published [7,13,14] and presented in conference [15] was carried out by the National Personal Protective Technology Laboratory of the National Institute for Occupational Safety and Health (NIOSH) to examine the cardiovascular and respiratory impact of EARL in pregnancy. This information could be useful to wearers of various facial coverings, respiratory protection program managers, exercise physiology researchers, sports medicine practitioners, and obstetricians.

## Materials and methods

Study participants included 32 healthy, non-smoking women of whom 16 were pregnant (13–35 weeks of gestation) and currently under the care of an obstetrician or licensed nurse midwife. Demographic characteristics of non-pregnant subjects were (mean ± standard deviation): age 24.8 ± 2.5 yrs, height 167.9 ± 6.3 cm, weight 66.0 ± 8.6 kg, Body Mass Index (BMI) 23.4 ± 3.0 kg/m^2^. For the pregnant subjects, these values were: age 28.1 ± 3.3 yrs, height 166.6 ± 6.2 cm, weight 69.0 ± 13.1 kg, Body Mass Index (BMI) 24.6 ± 3.8 kg/m^2^, and mean gestational age of 21.6 ± 4.7 wks. All study subjects were evaluated by a licensed physician prior to study participation and the study was carried out in a NIOSH research laboratory with mean temperature of 20.5°C and relative humidity of 42.1%. The study was approved by the NIOSH Human Subjects Review Board and all subjects gave verbal and written consent to participate.

Study subjects were randomized to wearing one of two models of N95 filtering facepiece respirators (N95 FFR) available as a one-size 3 M 9210 flat-fold model (3 M, St. Paul, MN ) or a Moldex cup-shaped model in either medium/large size (Moldex 2200) or small size (Moldex 2201) (Moldex, Culver City, CA). The EARL (filter resistances [pressure drop]) of the aforementioned three respective N95 FFRs were 94.1 Pa (9.6 mm H_2_O), 113.7 Pa (11.6 mm H_2_O) and 119.6 Pa (12.2 mm H_2_O) pressure when machine tested at 85 L/min of continuous airflow. The static dead space of the three N95 FFR models was, respectively, 375 ml, 280 ml, and 210 ml. To insure adequacy of N95 FFR fit and minimization of inward leakage, quantitative respirator fit testing was carried out utilizing the Portacount Plus® (TSI, Shoreview, MN) that counts the number of ambient airborne particles and particles within the deadspace of the N95 FFR to determine their ratio, termed the “fit factor”. A fit factor of ≥100, indicative of ≤1% entry of particles into the N95 FFR wearer’s breathing zone, is the minimum passing score on an Occupational Safety and Health Administration (OSHA) fit test (OSHA, 1998) and was required for study participation. A passing score was achieved by 22 subjects with the 3 M 9210 model, 7 subjects with the Moldex 2200 model, and 3 subjects with the Moldex 2201 model. Cardiovascular parameters (heart rate [HR], stroke volume [SV], cardiac output [CO], systolic blood pressure [SBP], diastolic blood pressure [DBP], mean arterial pressure [MAP], total peripheral resistance [TPR]) were obtained with the FinoMeter Pro® (FinaPres Medical Systems, Amsterdam, NL) that analyzes waveform data obtained via a finger cuff during continuous measurement of finger arterial pressure utilizing volume-clamp methodology [16]. The volume clamp method, as used in the FinoMeter, utilizes a cuff (clamp) with an inflatable bladder around the finger to maintain the diameter of a finger artery constant despite changes in arterial pressure with each heartbeat. With the volume of the artery then fixed, the pressure difference across the arterial wall (transmural pressure) is zero and intraarterial equals extraarterial pressure. The FinoMeter cuff contains an infrared plethysmograph that detects arterial volume changes with each heartbeat and infrared arterial pulsatile diameter changes that are used to alter the pressure in the cuff as needed to maintain the fixed arterial volume. The cuff pressure then serves as an indirect measure of intraarterial pressure [16]. Utilizing the ModelFlow methodology, stroke volume is determined by a three-element model using aortic characteristic impedance, arterial compliance, and systemic vascular resistance [17] and cardiovascular parameters obtained from the ModelFlow methodology are appropriately referred to as indexes. A finger cuff of appropriate size was positioned on the middle finger of the left hand and hydrostatic height correction was used to correct for the hand’s position with respect to the level of the heart. Pulse-derived oxygen saturation (SpO_2_) and transcutaneous carbon dioxide levels (TcpCO_2_) were continuously monitored with the Tosca 500 sensor (Radiometer, Copenhagen, DK) a combination heated (42°C) pulse oximeter and CO_2_ sensor attached to the right earlobe. Fetal heart rates (FHR) (n = 12) were obtained by the research physician with a Bidop ES-100 V3 ultrasound fetal Doppler (Koven Technology Incorporated, St. Louis, MO, US). After instrumentation, subjects donned the N95 FFR as per the manufacturer’s recommendation and performed a user seal check (OSHA, 1998) to evaluate the seal of the respirator to the face. The order of the trials (wearing an N95 FFR and controls) not wearing an N95 FFR was randomized; each trial and control consisted of three contiguous (non-randomized) 20 min phases of standing upright, pedaling a Kettler RX7 reclining bicycle ergometer (Ense-Parsit, North Rhine-Westphalia, DE) at 60 pedal cycles/min and 50 watts resistance, and sitting upright in a chair. Pregnant subjects were instructed to perform stationary walking for 30 seconds every 5 minutes during the standing portion of the trials and controls to prevent venous pooling in the lower extremities. Subjective impressions of exertion were obtained utilizing the Borg Rating of Perceived Exertion (RPE) that ranges from a rating of 6 (“very, very light”) to 20 (“very, very hard”) [18]. FHRs (n = 12) were captured at the beginning and end of each seated and standing session (FHRs were not assessed during exercise bicycle ergometer testing due to motion artifact and were unable to be detected in four pregnant subjects). There was a minimum 30 min respite between trials during which the FinoMeter Pro was removed. Each subject completed all of her testing on a single day.

### Statistical analysis

All dependent variables were summarized in 1 min averages and arranged for 10 min intervals of each standing, exercise, and sitting phase for statistical analysis. Repeated measures ANOVA in a mixed design (two within-subjects factors [condition × time] and one between-subjects factor [pregnancy]) was carried out to determine the main effect of EARL over time together with the effect of pregnancy on the variables. FHR, collected only at the baseline and end of each of the phases, was analyzed using two-way repeated measures ANOVA (condition × time). Greenhous-Geisser correction was adopted for assumption of sphericity and a post-hoc pair-wise comparison with Bonferroni adjustment was carried out for a significant F-value. A statistical significance was accepted when p < 0.05 and all analyses were performed using a statistical software package (SPSS v.18, IBM, Somers, NY).

## Results

**Figure 1:**
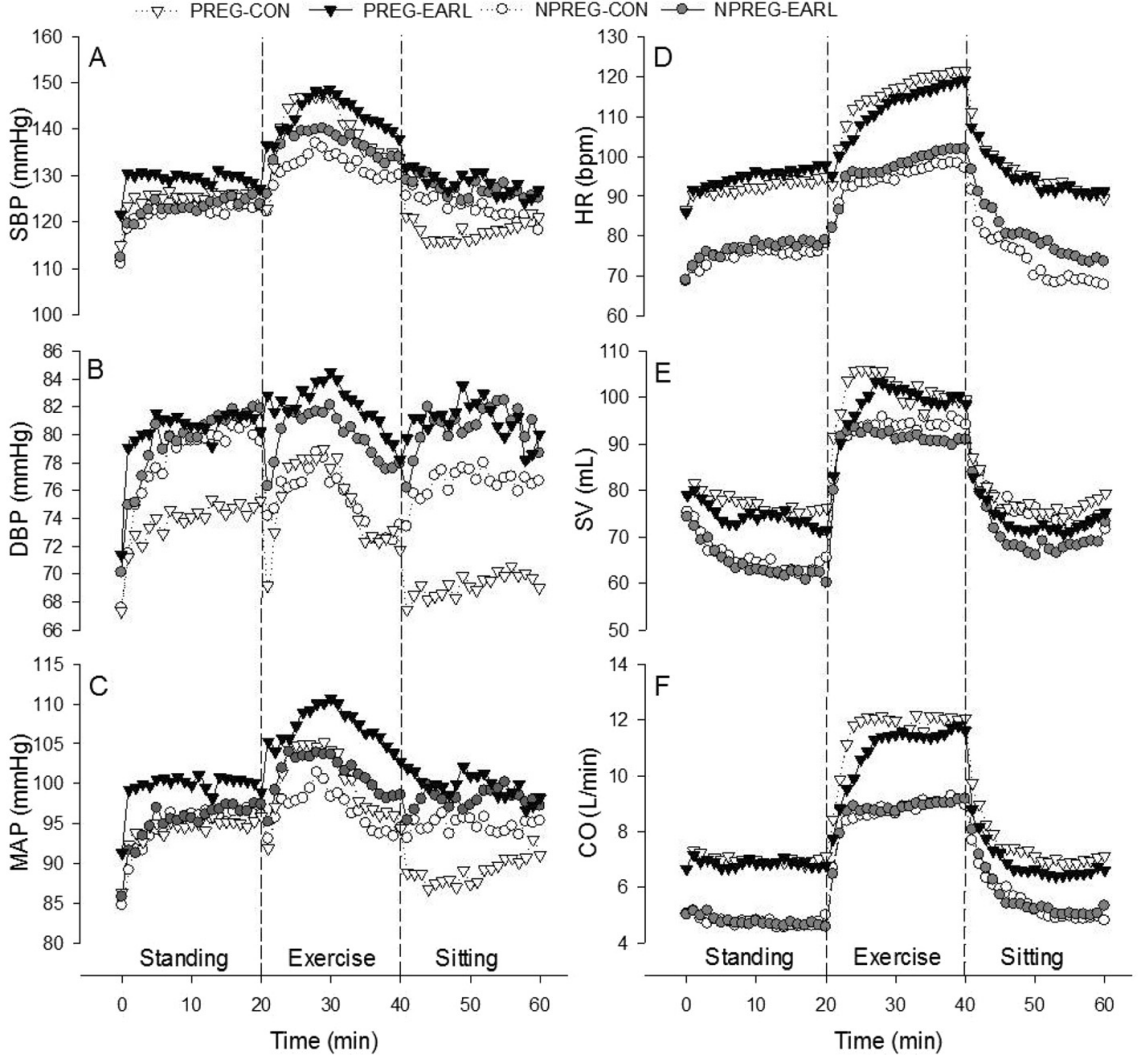
**Mean values for measured cardiovascular variables of pregnant controls (PREG-CON) and trials (PREG-EARL) and non-pregnant controls (NPREG-CON) and trials (NPREG-EARL).** Values are mean (n=32; 16 pregnant and 16 non-pregnant subjects). **(A)** SBP = systolic blood pressure, **(B)** DBP = diastolic blood pressure, **(C)** MAP = mean arterial pressure, **(D)** HR = heart rate, **(E)** SV = stroke volume, **(F)** CO = cardiac output.

**Figure 2:**
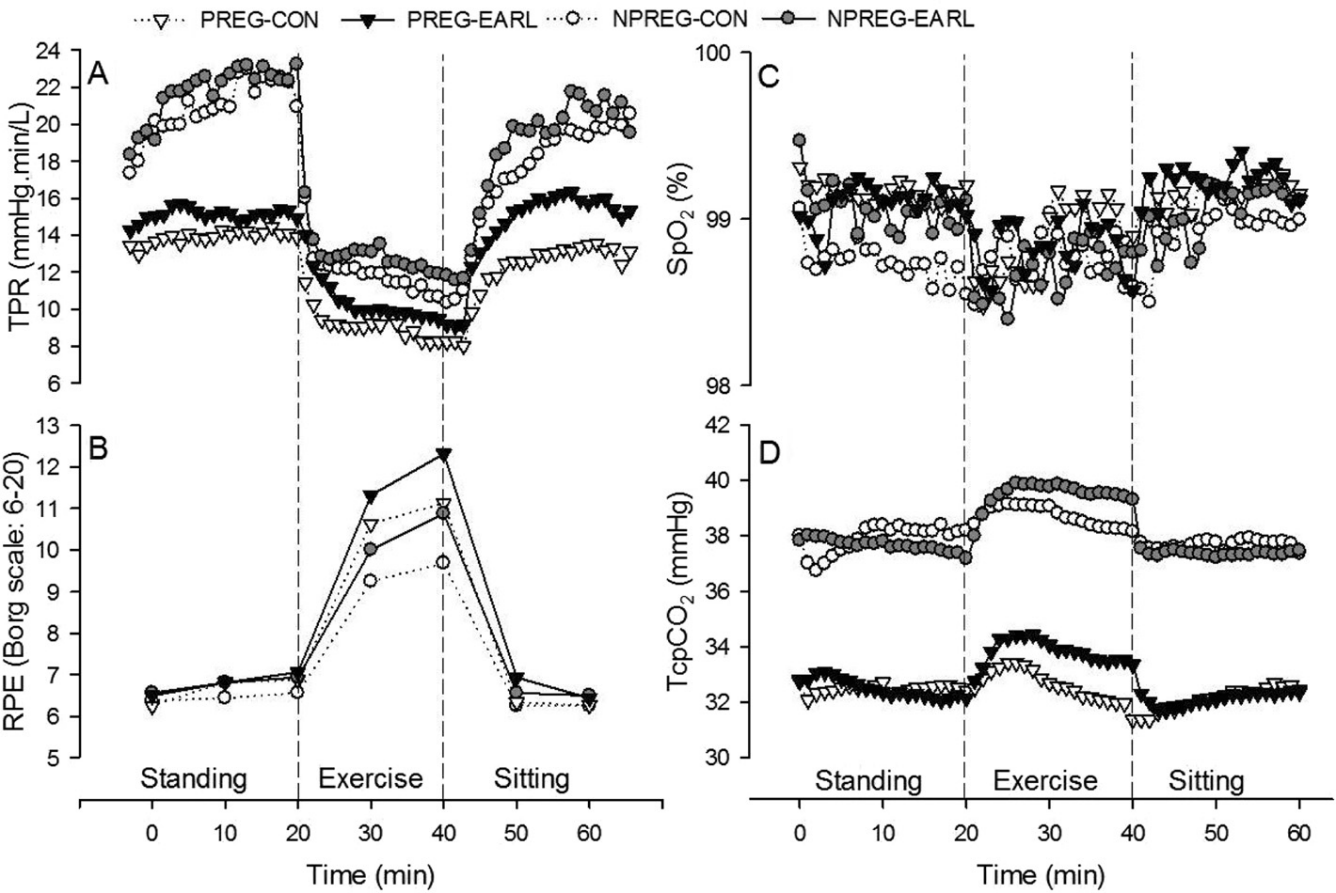
**Mean values for measured pulmonary variables, total peripheral resistance and subjective measures of exertion of pregnant controls (PREG-CON) and trials (PREG-EARL) and non-pregnant controls (NPREG-CON) and trials (NPREG-EARL).** Values are mean (n=32; 16 pregnant subjects and 16 non-pregnant subjects). **(A)** TPR = total peripheral resistance, **(B)** RPE = rating of perceived exertion, **(C)** SpO_2_ = pulse-derived oxygen saturation, **(D)** TcpCO_2_ = transcutaneous carbon dioxide.

Over the course of 1 h, the effects of EARL on vascular parameters included significantly elevated DBP (F = 9.198, p = 0.004) and MAP (F = 6.593, p = 0.01) on study subjects, but no significant differences in SBP (F = 3.763, p = 0.06) or TPR (F = 2.136, p = 0.15) (Figures 1A, B, C and 2A). None of the cardiac parameters was significantly impacted by EARL; HR (F = 1.916, p = 0.17), SV (F = 1.938, p = 0.17), CO (F = 0.414, p = 0.52) (Figures 1D, E, F), and FHR (F = 0.300, p = 0.59) (Table 1). Similarly, measured pulmonary variables of study subjects were not significantly different with EARL; SpO_2_ (F = 0.039, p = 0.84), TcpCO_2_ (F = 0.430, p = 0.83) (Figure 2C, D), but the RPE was significantly higher with EARL for all study subjects (F = 39.198, p < 0.001) (Figure 2B). No significant difference was noted for EARL in any measured physiological or subjective variable between the pregnant study group and the non-pregnant study group (Table 2). Time had no significant effect on FHR (F- = 1.798, p = 0.17), but had a significant effect on all other measured variables at the p < 0.001 level, save for SpO_2_ (F = 2.939, p = 0.04).

**Table 1:** **Fetal heart rates of 12 pregnant women, with and without applied external airflow resistive loads (EARL) of ~10 – 12 mm H**_2_ **O pressure**

**Trial**	**Stage (time in min)**			
	**Baseline (0)**	**End of standing (20)**	**End of exercise (40)**	**End of sitting (60)**
CONTROL	146.6 ± 11.3	144.5 ± 8.5	149.3 ± 13.8	148.5 ± 7.8
EARL	142.9 ± 6.8	143.7 ± 8.2	147.9 ± 7.0	150.1 ± 11.1

Values are mean ± SD (n = 12). No statistical difference between controls and trials.

**Table 2:** **Measured cardiovascular, pulmonary and subjective variables**

**Variables**			**SBP**	**DBP**	**MAP**	**HR**	**SV**	**CO**	**TPR**	**RPE**	**SpO2**	**TcpCO2**
**Subjects**	**Trial**	**Time**	**(mmHg)**	**(mmHg)**	**(mmHg)**	**(bpm)**	**(mL)**	**(l/min)**	**(mmHg · min/L)**	**(6-20)**	**(%)**	**(mmHg)**
Pregnant (n = 16)	CON	0	114.8 ± 13.5	67.3 ± 7.6	86.3 ± 10.6	86.5 ± 16.3*	78.9 ± 19.6	6.6 ± 1.5*	13.4 ± 2.6*	6.2 ± 0.4	99.3 ± 0.6	32.8 ± 2.0*
		10	125.4 ± 14.4	74.0 ± 7.7	94.8 ± 9.9	92.0 ± 14.0*	77.2 ± 15.7	6.9 ± 1.3*	13.8 ± 2.3*	6.8 ± 1.3	99.1 ± 0.8	32.6 ± 1.7*
		20	127.2 ± 12.6	75.3 ± 8.5	95.9 ± 10.5	94.8 ± 14.1*	76.1 ± 16.7	7.1 ± 1.4*	13.9 ± 2.6*	6.8 ± 1.5	99.2 ± 0.6	32.4 ± 1.9*
		30	147.0 ± 10.5*	77.6 ± 6.0	104.1 ± 7.9	116.9 ± 13.2*	102.6 ± 19.6	11.9 ± 2.5*	9.1 ± 2.3*	10.6 ± 1.6	99.0 ± 0.9	32.6 ± 2.1*
		40	133.8 ± 13.1	71.7 ± 7.4	94.3 ± 10.2	95.0 ± 15.4*	99.5 ± 17.6	12.0 ± 2.3*	8.0 ± 1.3*	11.1 ± 1.8	98.8 ± .0.7	31.3 ± 2.8*
		50	116.1 ± 12.7*	69.1 ± 8.1*	87.3 ± 8.8*	121.4 ± 13.5*	75.2 ± 16.8	7.0 ± 1.8*	12.9 ± 2.8*	6.3 ± 0.4	99.2 ± 0.4	32.0 ± 2.1*
		60	120.9 ± 10.8	69.0 ± 7.5*	90.9 ± 8.5	89.4 ± 12.0*	79.3 ± 16.0	7.1 ± 1.2*	13.0 ± 2.2*	6.2 ± 0.4	99.1 ± 0.6	32.4 ± 2.1*
	EARL	0	121.5 ± 12.8^#^	71.3 ± 7.5	91.2 ± 8.5	85.9 ± 13.5^#^	78.9 ± 15.9	6.6 ± 1.4^#^	14.2 ± 3.0^#^	6.5 ± 0.8	99.1 ± 0.3	32.7 ± 2.3^#^
		10	129.5 ± 12.9	80.6 ± 5.6	99.7 ± 7.6	96.2 ± 17.9^#^	74.0 ± 19.3	6.8 ± 1.4^#^	15.1 ± 3.0^#^	6.8 ± 1.2	99.1 ± 0.4	32.3 ± 2.0
		20	127.0 ± 13.4	80.2 ± 8.8	98.9 ± 9.5	97.7 ± 15.7^#^	71.4 ± 14.5	6.7 ± 1.0^#^	14.9 ± 2.8^#^	7.0 ± 1.3	99.0 ± 0.5	32.0 ± 1.6^#^
		30	148.5 ± 16.7	84.4 ± 10.3	110.6 ± 11.7	114.6 ± 15.8^#^	101.5 ± 19.3	11.4 ± 2.1^#^	9.9 ± 2.1	11.3 ± 1.9	98.8 ± 1.2	34.0 ± 1.3^#^
		40	137.9 ± 17.7	78.2 ± 9.9	102.7 ± 12.7	119.1 ± 17.1^#^	98.5 ± 21.5	11.6 ± 2.5^#^	9.1 ± 1.7	12.3 ± 1.5	98.5 ± 1.4	33.3 ± 1.5^#^
		50	129.0 ± 20.6	82.0 ± 14.9	100.9 ± 16.3	94.2 ± 14.2^#^	71.7 ± 15.8	6.6 ± 1.4^#^	15.8 ± 3.9	6.9 ± 1.1	99.1 ± 0.6	32.1 ± 1.5^#^
		60	126.8 ± 11.2	80.0 ± 8.1	98.2 ± 8.6	91.1 ± 14.0^#^	75.2 ± 14.8	6.6 ± 1.2^#^	15.3 ± 3.2^#^	6.4 ± 0.6	99.1 ± 0.6	32.3 ± 1.7^#^
Non-Pregnant (n = 16)	CON	0	111.0 ± 10.3	67.5 ± 6.3	84.7 ± 6.9	68.7 ± 11.1*	75.4 ± 19.5	5.0 ± 1.0*	17.3 ± 3.3*	6.3 ± 0.8	99.0 ± 0.6	38.0 ± 3.9*
		10	123.8 ± 14.0	79.5 ± 7.8	96.2 ± 7.9	76.8 ± 14.8*	64.5 ± 15.4	4.8 ± 1.0*	20.7 ± 4.3*	6.4 ± 0.6	98.7 ± 1.0	38.2 ± 4.8*
		20	123.5 ± 16.0	79.5 ± 9.1	96.0 ± 9.6	78.1 ± 15.6*	65.5 ± 19.6	4.9 ± 1.7*	20.9 ± 6.0*	6.5 ± 0.8	98.5 ± 1.8	38.1 ± 5.1*
		30	134.1 ± 15.8*	76.5 ± 10.3	98.3 ± 10.1	93.9 ± 15.6*	93.7 ± 23.3	8.6 ± 1.9*	11.9 ± 2.8*	9.2 ± 2.5	99.0 ± 0.6	39.0 ± 3.8*
		40	130.1 ± 12.2	73.5 ± 10.4	94.4 ± 10.8	97.1 ± 19.3*	94.1 ± 22.5	8.9 ± 2.0*	11.0 ± 2.5*	9.6 ± 2.9	98.5 ± 1.3	38.1 ± 3.4*
		50	124.3 ± 9.8*	77.5 ± 8.3*	95.6 ± 8.6*	70.1 ± 15.8*	76.2 ± 18.0	5.1 ± 1.0*	19.0 ± 3.2*	6.2 ± 0.4	99.0 ± 0.7	37.7 ± 3.4*
		60	118.2 ± 9.7	76.6 ± 7.2*	95.3 ± 9.4	67.8 ± 12.5*	71.6 ± 15.8	4.7 ± 1.1*	20.5 ± 3.5*	6.2 ± 0.4	98.9 ± 0.7	37.3 ± 3.3*
	EARL	0	112.3 ± 10.3^#^	70.1 ± 12.4	85.7 ± 10.5	69.0 ± 12.2^#^	74.3 ± 19.0	5.0 ± 1.2^#^	18.3 ± 6.3^#^	6.5 ± 1.0	99.1 ± 0.5	38.4 ± 4.1^#^
		10	122.9 ± 16.6	79.7 ± 14.0	95.6 ± 16.0	78.6 ± 14.6^#^	62.9 ± 17.8	4.7 ± 1.0^#^	21.5 ± 8.9^#^	6.8 ± 1.1	99.1 ± 0.5	35.4 ± 10.0
		20	123.8 ± 19.4	81.8 ± 13.7	97.3 ± 16.7	78.9 ± 15.5^#^	60.1 ± 18.7	4.5 ± 1.1^#^	23.2 ± 10.6^#^	6.9 ± 1.1	99.1 ± 0.5	37.4 ± 3.5^#^
		30	139.5 ± 20.2	82.1 ± 14.0	103.6 ± 17.3	97.3 ± 15.0^#^	90.9 ± 26.3	8.7 ± 2.3^#^	13.5 ± 8.4	10.0 ± 2.3	98.7 ± 0.7	39.7 ± 3.3^#^
		40	133.9 ± 19.7	77.9 ± 11.8	98.5 ± 15.6	101.9 ± 15.0^#^	90.9 ± 22.7	9.1 ± 2.2^#^	11.6 ± 5.4	10.8 ± 2.7	98.7 ± 0.6	39.3 ± 3.2^#^
		50	124.2 ± 13.7	79.2 ± 11.3	96.4 ± 12.6	79.5 ± 14.6^#^	68.7 ± 17.4	5.4 ± 1.5^#^	19.5 ± 7.3	6.5 ± 0.8	99.1 ± 0.6	37.2 ± 3.1^#^
		60	125.1 ± 14.7	78.6 ± 10.6	97.2 ± 12.4	73.5 ± 10.6^#^	73.2 ± 19.7	5.3 ± 1.4^#^	19.5 ± 6.0^#^	6.5 ± 1.0	99.1 ± 0.6	37.4 ± 3.0^#^

Values are mean ± SD (n = 16 pregnant and 16 non-pregnant).

*Significant difference between pregnant and non-pregnant CON trial (p < 0.05).

^#^Significant difference between pregnant and non-pregnant EARL trial (p < 0.05).

## Discussion

### Cardiac parameters

EARL did not result in a significant HR difference in study subjects (F = 1.916, p = 0.17). Baseline HR was higher in pregnant subjects (Figure 1D, Table 2), as is the norm in pregnancy due to diminished vagal tone [19], but the increase in HR with exercise is similar in pregnant women and controls, taking into account the elevated resting HR of pregnancy [20]. Recent data has indicated no significant difference in HR between mid-trimester pregnant women and non-pregnant controls with respect to orthostatic challenges [21] that is most likely related to the stabilizing effect of the increased blood volume after the first trimester [22]. The greater SV of pregnant subjects, as observed in the present study (Figure 1E, Table 2), is a normal finding in pregnancy due to increased end-diastolic volume and enhanced cardiac contractility [23], but EARL had no significant effect upon SV of study subjects (F = 1.938; p = 0.17). EARL was associated with a mean decrement in SV of ~7 mL that was equivalent in pregnant and non-pregnant subjects (Figure 1E). This slight decrease in SV might be related to the fact that EARL may induce a compensatory increase in the respiratory duty cycle (ratio of inspiratory duration to the total breathing cycle) [7] that results in pleural pressure changes and decreased left ventricular SV [24]. CO is normally higher in pregnancy (Figure 1F, Table 2) because it is the product of the increased HR and SV of pregnancy [10]. EARL had no significant effect on HR or SV of study subjects, so it is therefore not surprising that CO was correspondingly not significantly impacted (F = 0.414, p = 0.52). The normal FHR boundaries of 120–160 beats-per-minute [25] were not exceeded in the current study (Table 1) and the application of EARL on pregnant subjects had no significant FHR impact (F = 0.300, p = 0.59). Studies of the effect of maternal exercise upon FHR have provided variable findings, but investigations of healthy pregnant women (without EARL) at activity levels similar to the current study have reported similar FHRs [26,27], suggesting that EARL had little-to-no impact on FHR.

### Cardiovascular parameters

The effect of EARL on the SBP of study subjects approached, but did not achieve, statistical significance (F = 3.763, p = 0.06), but the interaction of time and pregnancy was significant for SBP (F = 2.645, p = 0.03). Although the similarity in SBP among pregnant and non-pregnant subjects (Figure 1A, Table 2) goes against the long-held dogma of decline in SBP from second to mid-third trimester, a recent prospective investigation with serial blood pressure measurements (sitting, standing) has documented a progressive rise in SBP during pregnancy without a mid-trimester drop [28]. DBP of study subjects was significantly higher (F = 9.198, p = 0.004) with EARL (Figure 1B, Table 2) and we assume that this is not an instrument artifact effect given that FinoMeter-measured DBPs have met the Association for the Advancement of Medical Instrumentation criteria for accuracy [29]. In support of our findings, an increase in DBP (p = 0.05), without concurrent significant increase in the SBP (p = 0.71), at comparable EARL levels (98.0 Pa [10 mm H_2_O] pressure) has previously been reported [30]. Further, a study evaluating the physiological effects of wearing a disposable filtering facepiece respirator (similar to N95 FFRs used in the current study) noted an increase in DBP, but not SBP, at a moderate work rate [31]. Breathing against resistance has been shown to influence inspiratory-related changes in intrathoracic pressure that impact venous return and SV, thereby influencing blood pressure responses [32]. Thus, although little research exists on the effects of respirator usage on blood pressure [31], there is some evidence [30,31] that EARL can impact DBP, though the physiological mechanism is currently not fully elucidated. The significant increase in DBP noted for all subjects in the current study with the addition of EARL might be of concern in pregnant subjects with preeclampsia, as it could have an additive effect on blood pressure that might be deleterious. A definitive answer to this issue would require a study of EARL in pregnant women with preeclampsia. EARL had a significant impact (F = 6.593, p = 0.01) on study subjects’ MAP (Figure 1C, Table 2) a not unexpected finding given that MAP (calculated as ([2 x DBP] + SBP) / 3) is influenced more by the DBP than the SBP, and the significantly increased DBP noted in the current study resulted in a significantly increased MAP. The TPR (calculated in abbreviated form as TPR ≅ MAP / CO) was lower in pregnant subjects (Figure 2A, Table 2), a normal observation in pregnancy due to the development of a low resistance uteroplacental vascular bed and the effects of circulating vasodilators (e.g., prostacyclin, progesterone, etc.) [10,28], but EARL had no impact on subjects’ TPR (F = 1.665; p = 0.20).

### Pulmonary variables

EARL had no significant effect on SpO_2_ (F = 0.039, p = 0.84) Figure 2C, Table 2), despite the fact that N95 FFR deadspace oxygen has been shown to be below ambient levels, because these levels are consistent with arterial oxygen that corresponds to SpO_2_ levels of 95% on the oxygen-hemoglobin dissociation curve [13]. TcpCO_2_ levels were lower in pregnant subjects due to the normally enhanced ventilation rate of pregnancy that allows for greater elimination of CO_2_ required to maintain a gradient that favors fetal-to-maternal CO_2_ transfer for elimination [14], but EARL had no significant effect (F = 0.430, p = 0.83; Figure 2D, Table 2).

### Subjective parameter

EARL has a significant effect on the RPE (F = 39.198, p < 0.001) on all subjects that was most evident during the exercise phase of the study (Figure 2B, Table 2). The increased airflow requirements of exercise, compared with those of sedentary states, result in increased resistance of filter material that may have been responsible for some of the subjective response [7]. Additionally, psychophysiological effects related to the thermosensitivity of the portion of the face covered by an N95 FFR, increased temperature of the inspired air, and claustrophobic or anxiety provoking actions of N95 FFR on some individuals may also have been operant to some degree in the RPE responses [33].

In summary, EARL (94.1 Pa [9.6 mm H_2_O] – 119.6 Pa [12.2 mm H_2_O]) itself did not have a significant impact on the majority of measured variables (HR, SV, CO, FHR, SBP, TPR, SpO_2_, PtcCO_2_) over the course of one hour of mixed sedentary (postural) and exercise activities in study subjects, but did have a significant effect on DBP (F = 9.198, p = 0.004) that influenced MAP (F = 6.593, p = 0.01), and on RPE (F = 39.198, p < 0.001). Pregnancy itself had no significant additional impact on any measured variables, but time had a significant effect on all measured variables except FHR. It is likely that, given the lower resistance levels of various fabrics used for facial coverings [4], and the fact that some facial coverings likely do not adhere as tightly to the face as N95 FFR, their impact on the wearers would be less than that noted in the current study for N95 FFR. However, the accumulation of significant amounts of expired moisture or sweat on facial coverings that adhere closely to the facial skin (e.g., niqab, balaclava, medical/surgical mask, etc.) could result in significant breathing resistance [34] that might equal that of some filtering facepiece respirators.

Limitations of the current study include the relatively small number of participants (n = 32); however, all study participants were experienced N95 FFR users, so that the observed physiological and subjective responses would not be influenced by lack of familiarity with the respirators. The study trials were carried out for one hour periods, so that we cannot comment on the impact of similar levels of EARL over longer periods of time. Due to not having pre-study data, it is possible that the SBP and DBP reported are higher than the subjects’ normal baseline due to any of the possible effects of relatively prolonged wear of the FinoMeter on the finger (i.e., interstitial fluid loss from the finger tissue under the cuff, altered blood flow, edema, or altered finger temperature at the measurement site [35,36]. FHR are sometimes not recordable for various reasons (e.g., presentation relatively early in gestation, fetal positioning, obesity, etc.) and were unable to be recorded for four of the current study’s pregnant subjects. The levels of EARL in the current study may be lower than the values obtained with machine testing at 85 L/min because human use of N95 FFRs results in variable face seal leakage that allows some air to bypass the filter and thereby decrease the resistance to airflow [7]. Various facial coverings (e.g., veils, balaclavas, niqabs, etc.) are loose fitting and the resistance to airflow is likely to be somewhat less than that of the tighter-fitting N95 FFR. For statistical analysis purposes, the study data were treated as one continuous variable over the course of one hour but significant differences might have existed if the individual phases of the study (i.e., standing, exercise, sitting) were analyzed separately. However, it was felt that the relative brevity of the individual phases (20 min) would not allow for clinically meaningful data. We cannot comment on EARL of higher or lower levels than those employed in the current study, nor on the effect (s) that might occur with EARL imposed for periods longer than 1 h. It is important to note that it is not only EARL that impacts those who wear facial coverings; associated issues of facial heat and humidity, increased perceptions of total body heat, variable carbon dioxide retention, and psychological issues (e.g., anxiety, claustrophobic reactions) must also be taken into consideration [33,37,38]. We tested subjective responses only for perceptions of exertion (RPE), but other subjective factors such as thermal sensations and psychological reactions (e.g., anxiety, claustrophobia) to wearing facial coverings could affect results. Although the static dead space volumes of the three N95 FFR models used in the current study differed by as much as 165 ml, the functional dead space that impacts breathing parameters could not be determined while the respirators were worn, so that no conclusions can be drawn with respect to dead space. Finger arterial pressure can overestimate brachial artery systolic pressure due to pulse pressure amplification that occurs with differences in vessel compliance between larger and smaller arteries [16]. Further, from the standpoint of comparisons of the impact of different respirator styles on measured variables, the significantly greater number of subjects passing fit testing with the 3 M 9210 (69%) precludes any meaningful comparisons with the small numbers for the Moldex 2200 (21%) and Moldex 2201 (10%) models.

## Conclusion

EARL that is imposed by various facial coverings with relatively low airflow resistance characteristics (i.e., 94.1 Pa [9.6 mm H_2_O] – 119.6 Pa [12.2 mm H_2_O]) may significantly impact some hemodynamic parameters (DBP, MBP) of pregnant and non-pregnant women alike during moderate exercise and sedentary activities, but the clinical significance of these effects is likely to be minimal over 1 h in healthy individuals. However, further investigation into the effects of EARL-related elevated DBP and MBP of pregnant women with preeclampsia is warranted. Subjective impressions of exertion (RPE) are heightened with EARL, notably during exercise, and may be due to the effect of increased airflow requirements on the resistance of the fabric covering the face and mouth. Pregnancy itself is not associated with significant differences of physiological and subjective responses (exertion) to the wearing of facial coverings with EARL at levels employed in the current study over 1 h at sedentary and moderate exercise activities.

## Abbreviations

BMI: Body mass index
CO: Cardiac output
DBP: Diastolic blood pressure
EARL: External airflow resistive loads
FHR: Fetal heart rate
HR: Heart rate
MAP: Mean arterial pressure
N95 FFR: N95 filtering facepiece respirator
NIOSH: National institute for occupational safety and health
OSHA: Occupational safety and health administration
RPE: Rating of perceived exertion
SBP: Systolic blood pressure
SpO_2_: Pulse-derived oxygen saturation
SV: Stroke volume
TcpCO_2_: Transcutaneous carbon dioxide
TPR: Total peripheral resistance
